# Using machine learning models to predict the initiation of renal replacement therapy among chronic kidney disease patients

**DOI:** 10.1371/journal.pone.0233976

**Published:** 2020-06-05

**Authors:** Erik Dovgan, Anton Gradišek, Mitja Luštrek, Mohy Uddin, Aldilas Achmad Nursetyo, Sashi Kiran Annavarajula, Yu-Chuan Li, Shabbir Syed-Abdul

**Affiliations:** 1 Department of Intelligent Systems, Jožef Stefan Institute, Ljubljana, Slovenia; 2 Executive Office, King Abdullah International Medical Research Center, King Saud bin Abdulaziz University for Health Sciences, Ministry of National Guard – Health Affairs, Riyadh, Kingdom of Saudi Arabia; 3 Taipei Medical University, Graduate Institute of Biomedical Informatics, College of Medical Science and Technology, Taipei, Taiwan; 4 Department of Nephrology, Yashoda Hospitals, Malakpet, Hyderabad, India; Universita degli Studi Magna Graecia di Catanzaro, ITALY

## Abstract

Starting renal replacement therapy (RRT) for patients with chronic kidney disease (CKD) at an optimal time, either with hemodialysis or kidney transplantation, is crucial for patient’s well-being and for successful management of the condition. In this paper, we explore the possibilities of creating forecasting models to predict the onset of RRT 3, 6, and 12 months from the time of the patient’s first diagnosis with CKD, using only the comorbidities data from National Health Insurance from Taiwan. The goal of this study was to see whether a limited amount of data (including comorbidities but not considering laboratory values which are expensive to obtain in low- and medium-income countries) can provide a good basis for such predictive models. On the other hand, in developed countries, such models could allow policy-makers better planning and allocation of resources for treatment. Using data from 8,492 patients, we obtained the area under the receiver operating characteristic curve (AUC) of 0.773 for predicting RRT within 12 months from the time of CKD diagnosis. The results also show that there is no additional advantage in focusing only on patients with diabetes in terms of prediction performance. Although these results are not as such suitable for adoption into clinical practice, the study provides a strong basis and a variety of approaches for future studies of forecasting models in healthcare.

## Introduction

In recent years chronic kidney disease (CKD) has reached a global prevalence as high as 11–13% with the majority in the stage 3. It is observed that the prevalence is higher for women than for men [1]. Asia records the highest prevalence worldwide, led by Japan and followed by Taiwan. A study showed that the overall prevalence of CKD stages 1–5 was 15.46% with an incidence of 27.21/1,000 people per year in Taiwan. The estimated average dwelling time was 5.37 years, which means that a person with CKD will either die or progress into the end-stage renal disease (ESRD) within 5 years [2]. The incidence of ESRD is rising with diabetic nephropathy accounting for nearly 45%. In 2015, the proportion of ESRD patients receiving haemodialysis and peritoneal dialysis is 87.5% and 8.5%, respectively [3]. The increase in ESRD populations worldwide is of a great concern for many countries as ESRD expenditures is consuming increasing proportions of the healthcare budget. In Taiwan, although ESRD patients represent only 0.15% of the total population, they are responsible for 7% of the total annual budget of Taiwan’s NHI Program owing to their use of dialysis [4, 5]. The prohibitive cost of renal replacement therapy (RRT), either dialysis or renal transplantation, makes it a huge burden for the health system. Often, CKD patients are not aware about their disease management, indicating a lack of education of patients about possible disease outcomes [2].

The RRT has been available in affluent countries for more than 50 years, with rapid growth in the number of people treated during this period. The use of dialysis to treat ESRD varies substantially between geographical regions, probably because of differences in population demographics, prevalence of ESRD, and factors affecting access to and provision of RRT [5, 6]. According to the 2016 United States Renal Data System (USRDS), 35.4% of ESRD patients do not receive adequate pre-ESRD care [7]. As a result, patients are often referred to a nephrologist when the disease gets complicated. Late referral is a strong factor for unplanned dialysis [8, 9]. Unplanned dialysis is defined as an emergency (life threatening) situation that occurs when a patient does not have a permanent access device (e.g., peritoneal catheter or arteriovenous fistula) yet [10]. However, if reliable indicators are made available for the doctors to forecast the necessity of dialysis in the foreseeable future, then both the doctor and the patient can prepare better for treatment in advance.

There is an ongoing debate among nephrologists regarding the optimal time to initiate dialysis for CKD patients. The Initiating Dialysis Early and Late (IDEAL) study was a randomised controlled trial study intended to investigate the influence of dialysis timing against mortality risk. This study revealed no differences between early and late dialysis towards mortality risk [11]. Escoli et al. study argued against early initiation of RRT citing higher mortality in these subset of patients [12]. Although there is no definitive evidence on the optimal time to initiate RRT, Kidney Dialysis outcomes Quality Initiative guidelines suggest to consider both the glomerular filtration rate (GFR) and clinical condition [13]. Although physicians and nephrologists are advised to closely monitor the decline in GFR of CKD patients, lack of infrastructure prevents many health care facilities from monitoring it [14].

Given quality data on dialysis, relevant information such as time to start dialysis can be predicted with Machine Learning (ML) algorithms. ML algorithms are a category of Artificial Intelligence (AI), which aims at simulating human intelligence by discovering patterns and using inference on the available data [15]. AI is being increasingly used to assist diagnosis, therapy, automatic classification and rehabilitation. For example, ML algorithms have been applied for the prediction of starting RRT within a few months to a year [16–20], and prediction of acute kidney injury that required RRT within a few hours to a few days [21–24]. However, the existing work uses laboratory (and demographic) data for the analysis and prediction of RRT. While the results also showed that laboratory data are the most potent predictors for RRT, they are—as mentioned before—not always available.

In this paper we explore the possibilities of using ML models to predict whether a CKD patient will require RRT (i.e., either to start dialysis or undergo transplantation) at some point in the next 3, 6, or 12 months, based only on the comorbidities of patients. While the patients whose data was used to train the models started RRT based on their clinical status and laboratory data, so the decision was made as well as possible, we try to approximate this decision with more limited data. The advantage of this approach is that it can be used on data easily extracted from hospital databases and that it does not rely on GFR or other laboratory data, which may be expensive for the health system to obtain several times in a row for patients in low- and medium-income countries. In addition, such models can be of high benefit for the policy-makers, hospital managers, and insurance companies, as they provide a better insight into the trends and thus allow for better allocation of resources.

### Objective

The objective of our study is to develop a screening tool based on disease history using various ML models to predict upcoming RRT at the time of CKD diagnosis, and to validate these models. A further goal is to identify what comorbidities (such as diabetes) play major role in the models that forecast the onset of RRT for different time periods. This tool may serve both medical doctors and CKD patients in better healthcare planning and management of resources.

## Methods

### Study design

**Fig 1:**
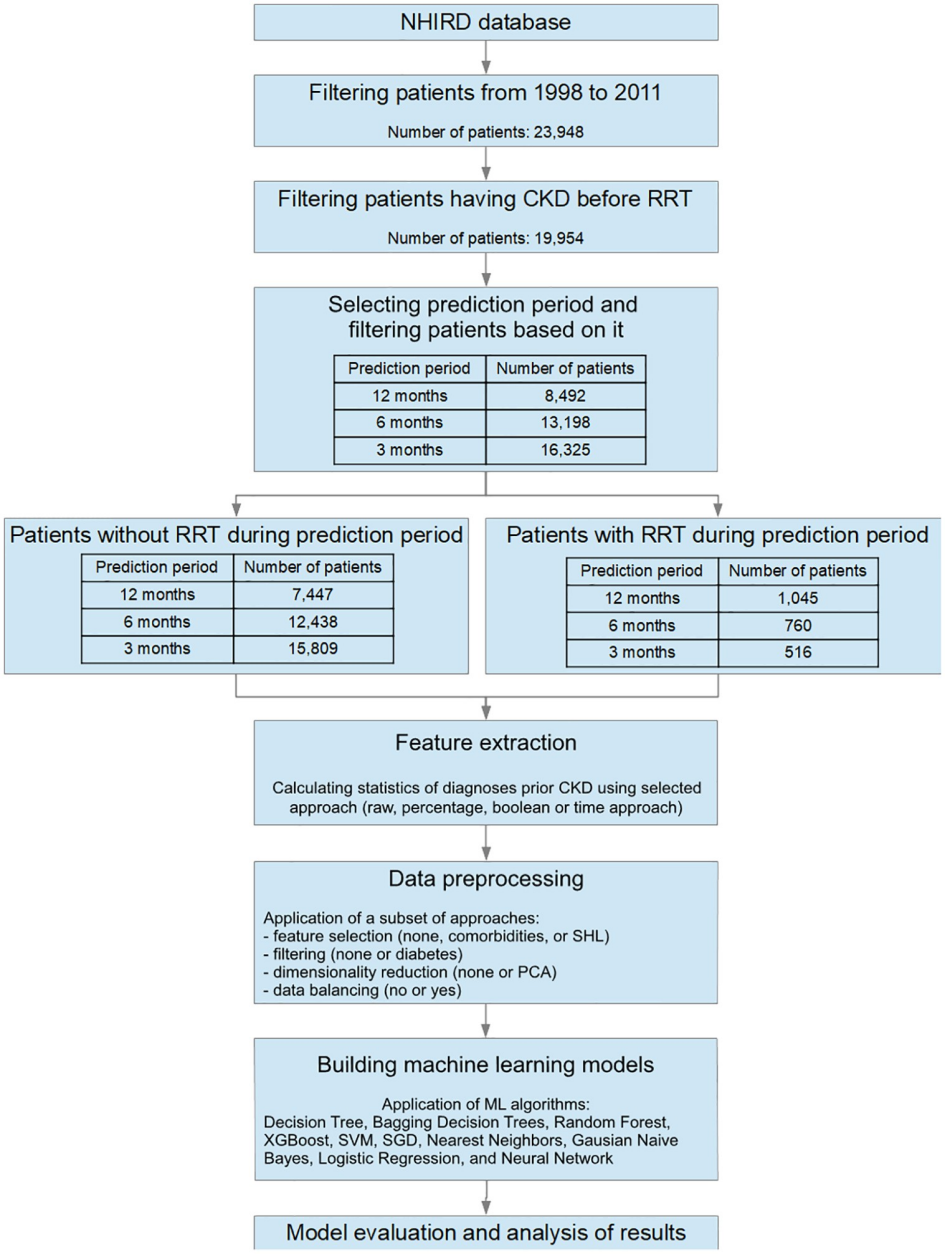
Data processing and model building procedure.

The study design is a retrospective study. A group with the outcome of interest (hemodialysis, peritoneal dialysis, or renal transplantation, i.e., RRT) within a time period of 3, 6, or 12 months after CKD diagnosis is matched with a control group who did not enter RRT within this time period. Retrospectively, the researcher determines individuals who were exposed to CKD diagnosis for the first time in each of the study groups. The procedure for processing the data and building the ML model is shown in Fig 1.

### Data source

The dataset is derived from Taiwan’s national health insurance research database (NHIRD) and consists of patients’ healthcare insurance information and claims data [25]. NHIRD is one of the largest and most comprehensive health insurance databases running under the Ministry of Health and Welfare of Taiwan. It includes data from primary outpatient departments and inpatient hospital care settings covering 99% of the Taiwanese population, i.e., approximately 23 million people, and is one of the most powerful data resources for biomedical research in that region. Among other data, NHIRD also contains diagnoses for each patient’s visit to the doctor. However, NHIRD does not contain laboratory values.

In our study, we analysed a subset of anatomised secondary data, i.e., from 1998 to 2011. This study has been exempted by the Institutional Review Board of Taipei Medical University beforehand.

### Patient selection

The selected subset of data included 23,948 patients. They were further filtered by selecting only those with the CKD diagnosis, which had to occur before RRT. The time of RRT was determined with the first occurrence of hemodialysis, peritoneal dialysis or renal transplantation. This resulted in 19,954 patients.

The patients were further filtered with respect to the time between the diagnosis of CKD and initiation of RRT. As mentioned, three intervals were tested: 3, 6, and 12 months. When the interval of *n* months was tested, we selected only those patients who had data for *n* months after they were diagnosed to have CKD. This filter ensured that the observed patients were labeled with the correct classes with respect to the entire observed period. The resulting number of patients for each interval is shown in Fig 1.

### Outcome definition

**Fig 2:**
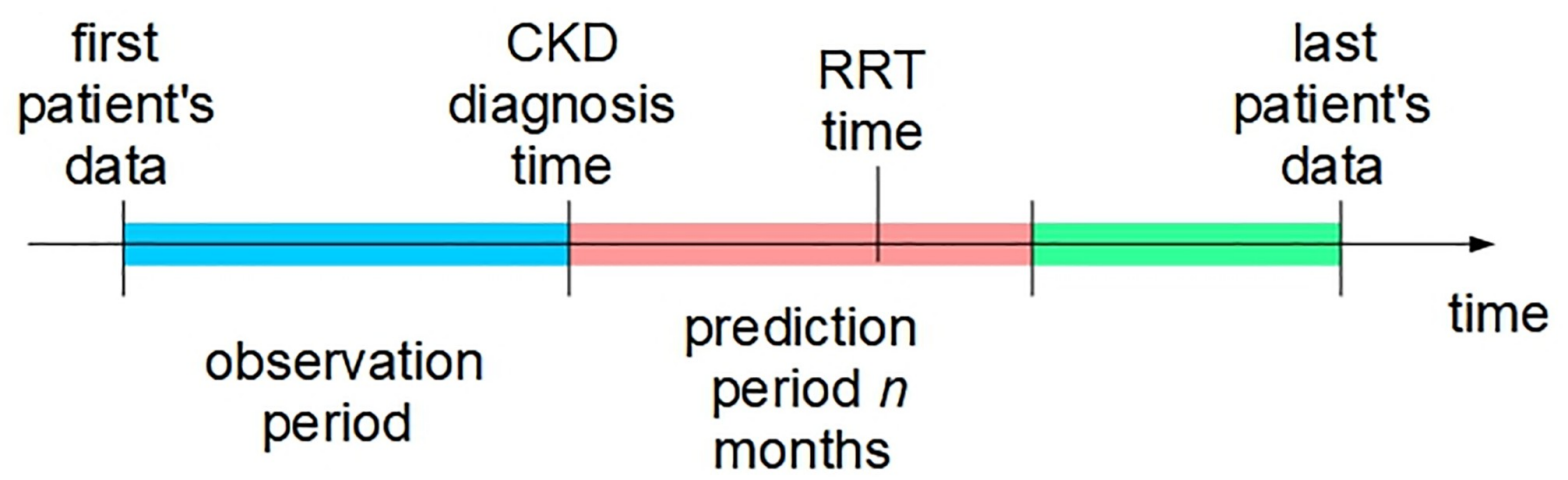
Relations between various time events used to define the RRT outcome.

The aim of this study is to predict the outcome (RRT) at the time of CKD diagnosis. We define the CKD diagnosis time as the day the patient is first diagnosed with CKD (ICD-9-CM code 585). Patients are then followed for the next *n* months with *n* equal to 12, 6, and 3 months (prediction period). For each of the three values of *n*, if RRT occurs within *n* months, the patient is labeled as 1, otherwise he/she is labeled as 0. When predicting the patient’s outcome, only data prior to CKD diagnosis time (observation period) is taken into account. Fig 2 shows the relations between outcome definition, and observation and prediction periods.

### Feature extraction

The data of the observation period include patient’s diagnoses recorded by the physician at each patient’s visit. Each diagnosis in the database represents a feature, where for each patient’s visit, the diagnosis is set to 1 if it was diagnosed, otherwise it is set to 0. Afterward, all the patient’s visits are combined together into a single machine-learning instance, resulting in one instance per patient. The visits are combined in four ways using raw, percentage, Boolean, and time approach.

- Raw approach: For each diagnosis, this approach counts the number of its occurrences by the time CKD was diagnosed.
- Percentage approach: For each diagnosis, we calculate the percentage of its occurrences with respect to all the patient’s visits.
- Boolean approach: Each diagnosis is set to 1 if it occured at least once, or 0 if it never occured.
- Time approach: This approach is similar to the Boolean approach. However, instead of setting just one Boolean value for the entire observation period, we divide the observation period into several subperiods. Then we determine whether the diagnosis occurred (1) or not (0) for each subperiod. These values are then weighted for all the observed subperiods and summed up, where higher weights are given to more recent periods.
  In this study, we divided the observation period into seven six-months subperiods and the remaining period, starting from the CKD diagnosis time. The weights for these periods were calculated as $w_{i}={\left(\frac{1}{2}\right)}^{i}$, where *i* = 1, …, 7 is the interval index, with *i* = 1 is being the most recent interval, and *i* = 7 being the most remote one. The remaining period had the same weight as the most remote interval. As a result, the sum of these weights is 1.

### Data preprocessing

The data were preprocessed with four methods in order to determine whether preprocessing could improve the results. More precisely, three methods, i.e., feature selection, filtering and dimensionality reduction, were individually applied before ML, while the fourth approach—data balancing—was implemented by the ML algorithms.

#### Feature selection

Three feature selection approaches were evaluated. The first approach consisted of calculating the correlations between diagnoses, i.e., features, and RRT. Then we removed the diagnoses with the absolute correlation lower than 0.1.

The second approach selected only those diagnoses that are related to CKD, i.e., comorbidities. The set of comorbidities was defined based on [26] and included acute glomerulonephritis, chronic glomerulonephritis, diabetes mellitus, essential hypertension, hyperlipidemia, polycystic kidney disease, renal stone, systemic lupus erythematosus, congestive heart failure, coronary artery disease, cerebrovascular disease, and proteinuria.

The third approach found the relevant diagnoses based on mutual information with class and contribution to Random Forest classifier performance, where diagnoses were sequentially added to the input data until the performance of the classifier stopped increasing. This method was part of the winning approach of the Sussex-Huawei Locomotion-Transportation (SHL) recognition challenge 2018 [27].

#### Filtering

The data analysis also included the selection of only those patients that had diabetes type 2 or type undefined before or at the time of CKD diagnosis. Such a filtering approach aimed at determining whether better ML models could be obtained if the data of a more homogeneous group of patients is used.

#### Dimensionality reduction

The dataset included 5,624 diagnoses, thus we had 5,624 features. The first approach to reduce this large number of features was feature selection as described above. In addition, we also evaluated an additional approach that reduced the number of features by combining them into a lower number of features. More precisely, we applied Principal component analysis (PCA), which is a statistical procedure that converts (possibly) correlated features into a set of values of linearly uncorrelated features, i.e., components. The first 50 PCA components, i.e., those with the highest variance, were selected for further analysis.

#### Data balancing

The dataset is very unbalanced with respect to the class, i.e., RRT. More precisely, the majority class, i.e., no RRT, represents 87% of data for the twelve-months prediction period, 94% for the six-months prediction period, and 96% for the three-months prediction period. To overcome this issue, data balancing should be used. In our experiments, we balanced the data by applying weights to instances, i.e., patients, where the weights were inversely proportional to class frequencies in the database.

### Model development

We evaluated 10 ML algorithms that are implemented in the Python packages Scikit-learn [28] and XGBboost [29]: Decision Tree, Bagging Decision Trees, Random Forest, XGBoost, Support Vector Machines, Simple Gradient Descendent, Nearest Neighbors, Gausian Naive Bayes, Logistic Regression, and Neural Network.

Decision Tree algorithm implements a tree structure, where each internal node represents a condition for a feature, each branch represents satisfaction of the node condition, and each terminal node determines the class assigned to the instances that met the conditions of the internal nodes on the path from the root node to the terminal node [30].

Bagging Decision Trees applies a set of decision trees, where each decision tree is built only on a random subset of data. The final classification is determined by the voting mechanism, i.e., the most voted prediction is the final prediction [31].

Random Forest upgrades Bagging Decision Trees with random feature selection. More precisely, when partitioning a node, a subset of features is randomly selected and only those features are considered during the partitioning [31].

Extreme Gradient Boosting (XGBoost) is an upgrade of Random Forest, where models (in our experiment, Decision Trees) are built sequentially to minimize the errors and maximize the influence of the best models. To minimize the errors, gradient descendent algorithm is applied. In addition, over-fitting avoidance mechanisms are applied such as tree-pruning and regularization [29].

Support Vector Machines (SVM) algorithm is a binary classifier that maps the input data in a very high-dimension feature space with a non-linear transformation (also known as the kernel trick), and applies a linear decision surface in the feature space to discriminate between the two classes [32].

Simple Gradient Descendent (SGD) is an algorithm that in the selected setup trains the linear SVM classifier. It also applies minibatches, regularizers and other mechanisms for avoiding over-fitting [33].

Nearest Neighbors classifier finds the nearest (closest) neighbors in the feature space by the means of Euclidean distance, and applies a majority vote on classes of these nearest neighbors to determine the class [34].

Gaussian Naive Bayes method applies Bayes’ theorem with the assumption of conditional independence between every pair of features given the class value [35].

Logistic Regression applies the logistic function to predict the probability of the default class in a two-class problem. It is a linear model, since the features are summed up linearly using weights, however, the predictions are transformed using a non-linear logistic function [36].

Neural Network model represents a (significant) enhancement of the logistic regression method. Similarly to logistic regression, it linearly combines the features and applies a (non-linear) transformation on the result. This is then upgraded by stacking several such transformations into layers, thus obtaining several hidden layers (in addition to the input, i.e., features’ layer, and the output, i.e., class layer), where each layer represents a different level of abstraction. In addition, several mechanisms can be applied to improve the performance and prevent overfitting, such as regularization, dropout, training in batches etc. [37].

The key parameter values of the applied algorithms are shown in Table 1. Note that the default parameter values have been used with respect to their implementation in Scikit-learn [38] and XGBboost [39] packages, with the exception of Neural Network, whose parameters were set as shown in Table 1.

**Table 1:** Parameter values of evaluated ML algorithms.

Algorithm	Key parameter values
Bagging Decision Trees	Number of trees = 10
Random Forest	Number of trees = 10
XGBoost	Number of trees = 100, max tree depth = 3, learning rate = 0.1
Support Vector Machines	Kernel = rbf, C = 1.0
SGD	Loss = log, penalty = l2, max iterations = 1000
Nearest Neighbors	Number of neighbors = 5
Logistic Regression	penalty = l2, max iterations = 100
Neural Network	Sizes of hidden layers = [100, 20], solver = adam, activation = relu, batch size = 10, max iterations = 200

## Experiments and results

### Experiment execution and performance metrics

Each experiment consisted of one ML algorithm and a combination of data preprocessing approaches (filtering, feature selection etc.). The evaluation was performed with stratified 10-fold cross-validation. This method split the dataset into 10 folds, where all the folds had the same ratio of RRT and non-RRT patients. Afterwards, the ML algorithm was applied 10 times, each time selecting a different fold for testing purposes and learning on the other nine folds.

As the main performance metric we selected the Area Under the Radio Operator Characteristic Curve (AUC). Note that the final AUC was the mean of AUCs obtained at all the folds. In addition to the AUC, we also observed sensitivity and specificity of the ML models. AUC measures how good is a model that classifies patients into two classes, for example, RRT and non RRT. AUC is also often used to estimate the optimal model’s threshold to be used in real situations [40]. Different healthcare providers in different situations may be interested in different trade-offs between sensitivity and specificity. AUC is an aggregate measure of a model’s quality across all such trade-offs. The ROC (which AUC is based on) allows one to select the desired trade-off, which corresponds to a threshold to be applied to the model’s output.

In the presented experiment, sensitivity measures the capability of the ML model to successfully predict RRT for patients who did in fact have RRT in the observation period. In a similar fashion, specificity shows the capacity of the ML model to correctly predict the absence of RRT for patients without RRT in the observation period [41]. Sensitivity and specificity have been defined by Yerushalmy [42] and have been used in medical literature since then.

### Results of ML algorithms predicting 12 months ahead

In this experiment we tested the effect of all the implemented data preprocessing approaches on the results of ML algorithms. Since the number of combinations of preprocessing approaches is large, we evaluated only the effect of one data preprocessing approach at a time. The only exception was data balancing, which was always tested, i.e., for each combination, we tested with and without data balancing.

**Fig 3:**
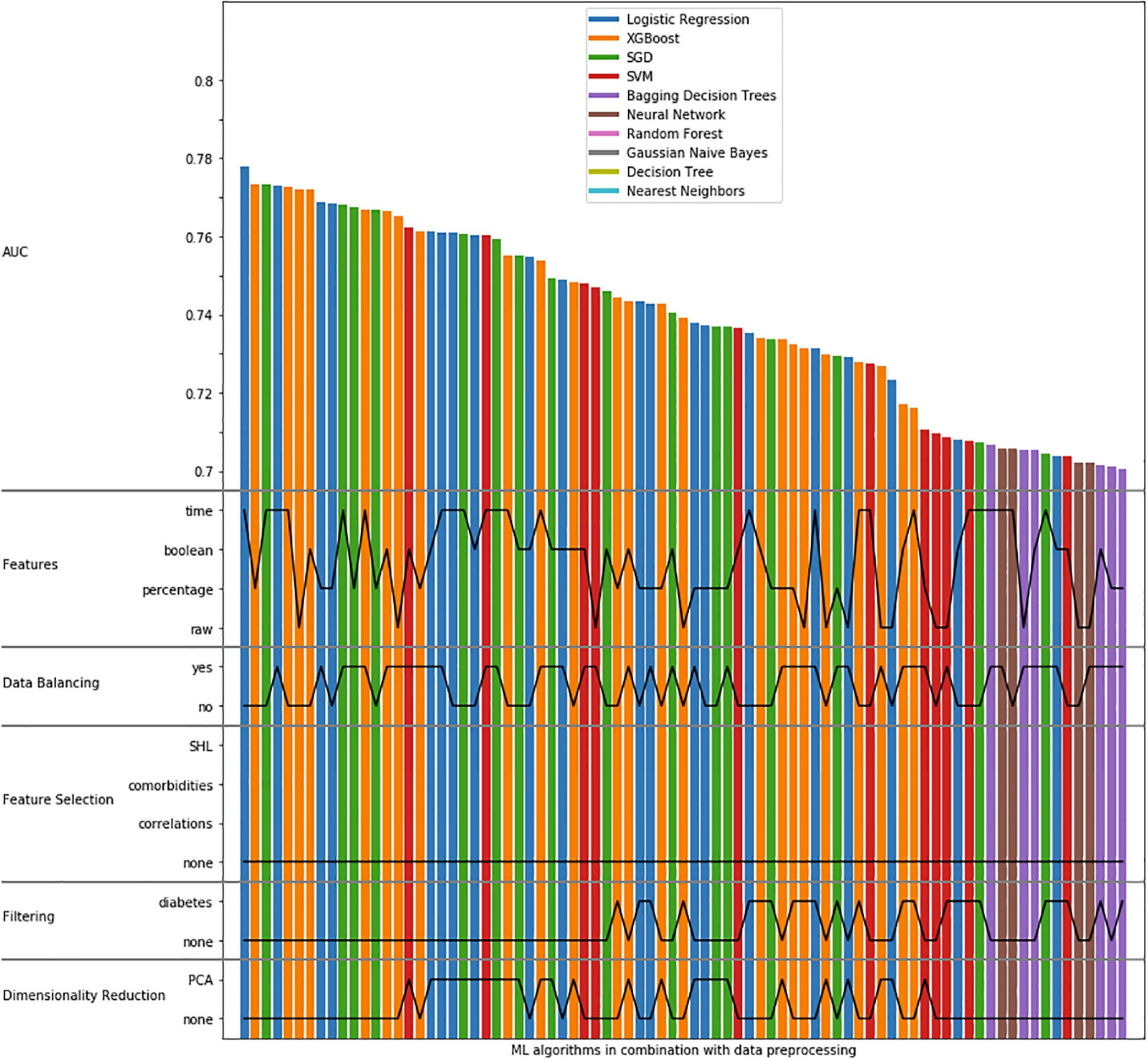
AUCs obtained with the ML algorithms with data preprocessing details, where only AUCs > 0.7 are shown.

**Fig 4:**
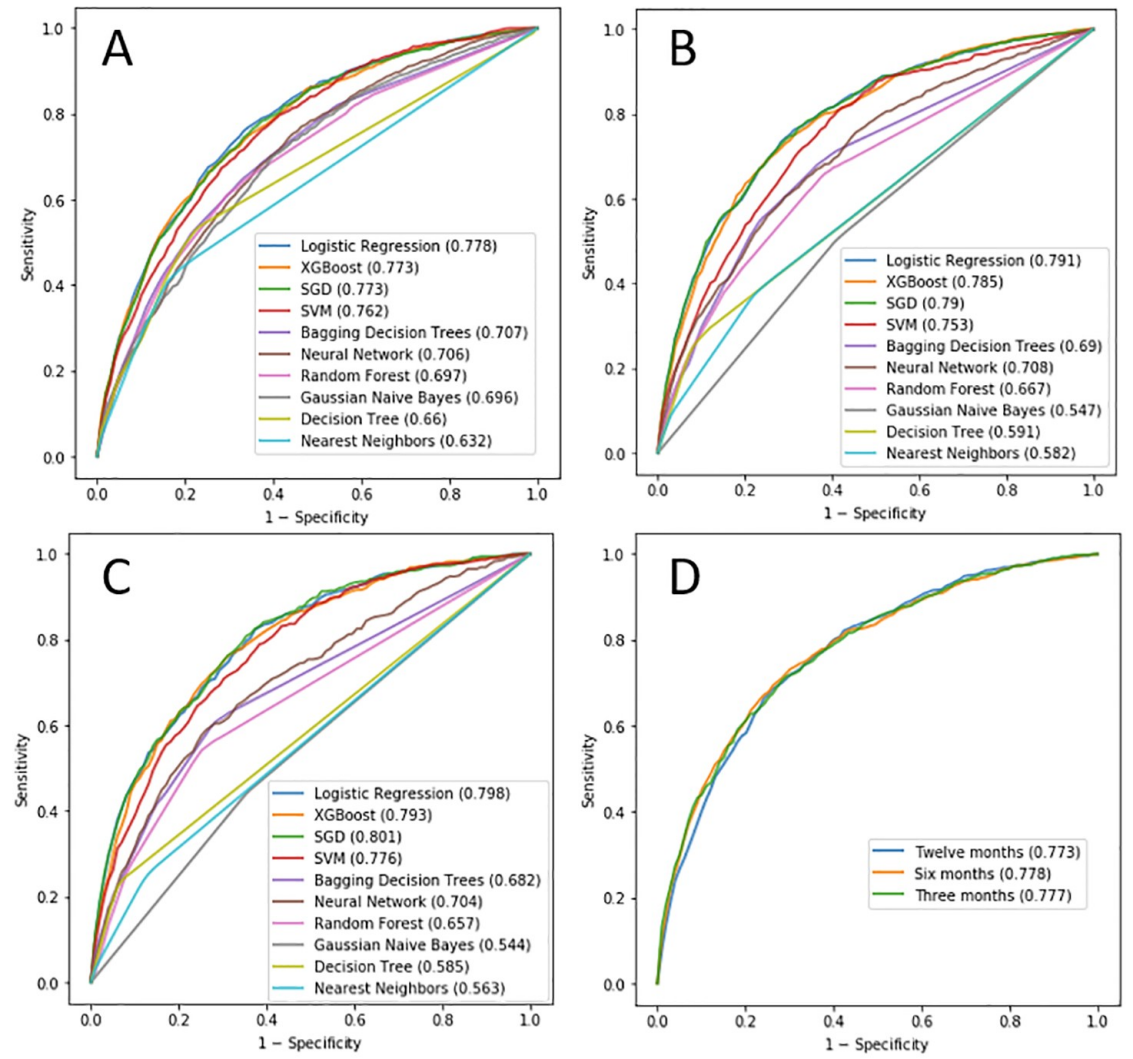
The best ROC curves of each ML model, obtained by testing various configurations in terms of feature extraction, data balancing, feature selection, filtering and dimensionality reduction, for A) 12 months, B) 6 months, C) 3 months. The AUC values are shown in brackets. D) AUCs obtained with Logistic Regression for various prediction periods.

The results were sorted with respect to the AUC and the top 10 results are shown in Table 2. Note that these top 10 results were obtained with no feature selection, no filtering, and no dimensionality reduction. In addition, Fig 3 shows the data processing parameters of the algorithms that obtained AUC > 0.7. On the horizontal axis, each bar represents an instance of ML algorithm in combination with data preprocessing. The vertical axis shows the AUC and data preprocessing parameters. More precisely, it shows from top to bottom: AUC, the used features, data balancing, feature selection, filtering and dimensionality reduction. The AUC values are shown with the bar height, while the data preprocessing parameters are shown with black (horizontal) lines. For example, the algorithm on the extreme left (AUC = 0.778) uses Logistic Regression, time features, no data balance and no feature selection, as well as no filtering and no dimensionality reduction. The ROC curves obtained with the ML algorithms are shown in Fig 4A.

**Table 2:** Top 10 algorithms in combination with data processing, when predicting twelve months ahead. Results are sorted with respect to AUC. These top 10 results were obtained with no feature selection, no filtering, and no dimensionality reduction.

Model	Features	Balance	AUC	Sensitivity	Specificity
Logistic Regression	time	no	**0.778**	0.129	0.983
XGBoost	percentage	no	0.773	0.057	0.993
SGD	time	no	0.773	0.145	0.978
Logistic Regression	time	yes	0.773	**0.623**	0.781
XGBoost	time	no	0.773	0.049	0.994
XGBoost	raw	no	0.772	0.036	0.995
XGBoost	Boolean	no	0.772	0.042	0.995
Logistic Regression	percentage	yes	0.769	0.581	0.794
Logistic Regression	percentage	no	0.769	0.0667	0.992
SGD	time	yes	0.768	0.583	0.796

The results show that the highest AUC is obtained when no feature selection, no filtering, and no dimensionality reduction is applied (Fig 3). While the highest AUC is achieved without data balancing, data balancing ensures a much more reasonable trade-off between sensitivity and specificity without reducing the AUC much (see, for example, the results of Logistic Regression in rows 1 and 4 in Table 2). Finally, features obtained with the time approach were the best ones.

These results are also confirmed by S1 Table–S5 Table in S1 File. These tables show statistics of AUC for each feature extraction approach, data preprocessing and ML algorithm independently. Note that to obtain a better trade-off between sensitivity and specificity, we decided to always apply data balancing.

### Results of ML algorithms predicting six and three months ahead

The procedure for the twelve-months-ahead prediction was also applied for six- and three-months ahead. However, we did not compare the approaches for feature selection, filtering and dimensionality reduction, since none of them were good for the twelve-months-ahead prediction. The ROC curves obtained with the ML algorithms when predicting six months ahead are shown in Fig 4B, while Fig 4C shows the ROC curves when predicting three months ahead.

The results show that, similarly to the twelve-months-ahead prediction, data balancing ensured better trade-off between sensitivity and specificity in comparison to when no balancing was used. In addition, the features obtained with the time approach were the best ones (see S6 Table–S7 Table in S1 File). The best ML models were Logistic Regression and SGD. Since Logistic Regression is simpler to interpret, we use it for further analysis.

### Further analysis with Logistic Regression

The best results were obtained with Logistic Regression in combination with time features and data balancing, and without feature selection, filtering or dimensionality reduction. The performance of Logistic Regression at various prediction periods including confusion matrices and ROC curves is shown in Table 3 and Fig 4D. In addition, confusion matrix of twelve-months model is shown in Table 4, while confusion matrices for six- and three-month models are shown in S8 Table–S9 Table in S1 File.

**Table 3:** Performance metrics for various prediction periods.

Prediction period	AUC	Sensitivity	Specificity
Twelve months	0.773	0.623	0.781
Six months	0.778	0.568	0.832
Three months	0.777	0.504	0.863

**Table 4:** Confusion matrix for twelve-months-ahead prediction.

		Predicted	
		No	Yes
True	No	5819	1628
	Yes	394	651

**Fig 5:**
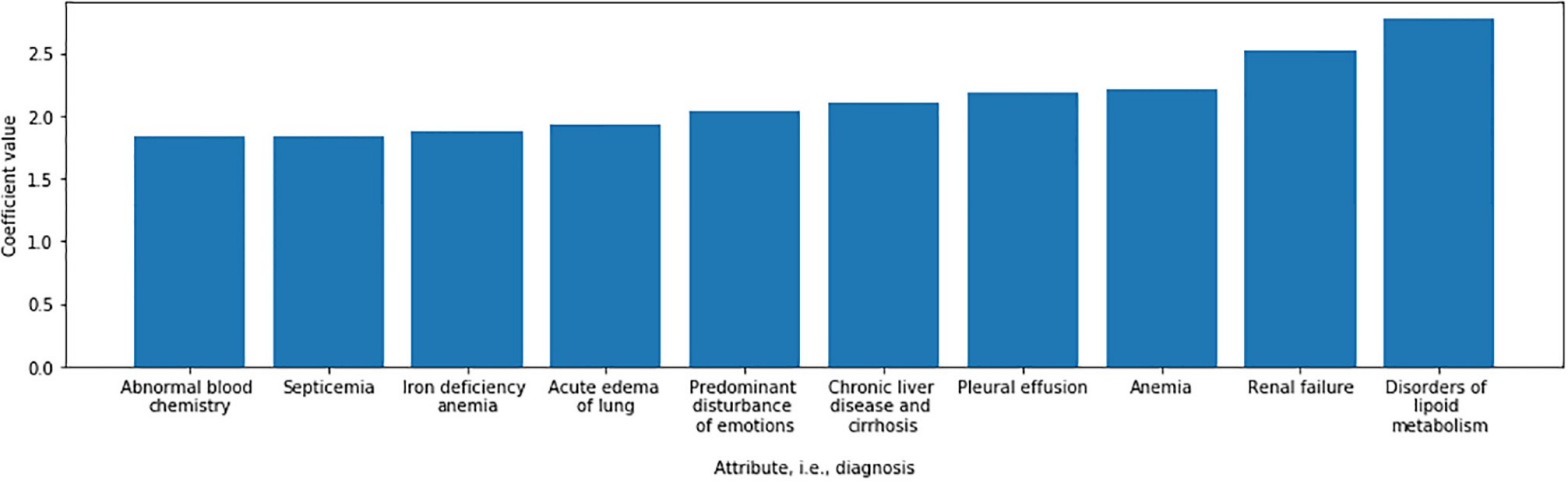
Diagnoses with the highest importance, i.e., coefficients, when applying Logistic Regression for twelve-months-ahead prediction.

Fig 5 shows the coefficients that are obtained when predicting twelve months ahead. Note that here we only show 10 most important features/diagnoses, i.e., those with the highest absolute coefficients. Some of these features, such as disorders of lipoid metabolism and renal failure are self explanatory in predicting, while others have a large overlap with a variety of diseases. For example, lipid abnormalities are common in patients with nephrotic syndrome in the form of hypercholesterolemia and hypertriglyceridemia. Some of these patients may as well be unresponsive to therapy and progress to CKD. In addition, CKD per se has hypertriglyceridemia. Hence lipid abnormalities are common in a variety of renal diseases which may progress to CKD. Almost all patients get emotionally disturbed once they get to know they have renal failure.

### Analyzing RRT probabilities obtained with Logistic Regression

**Fig 6:**
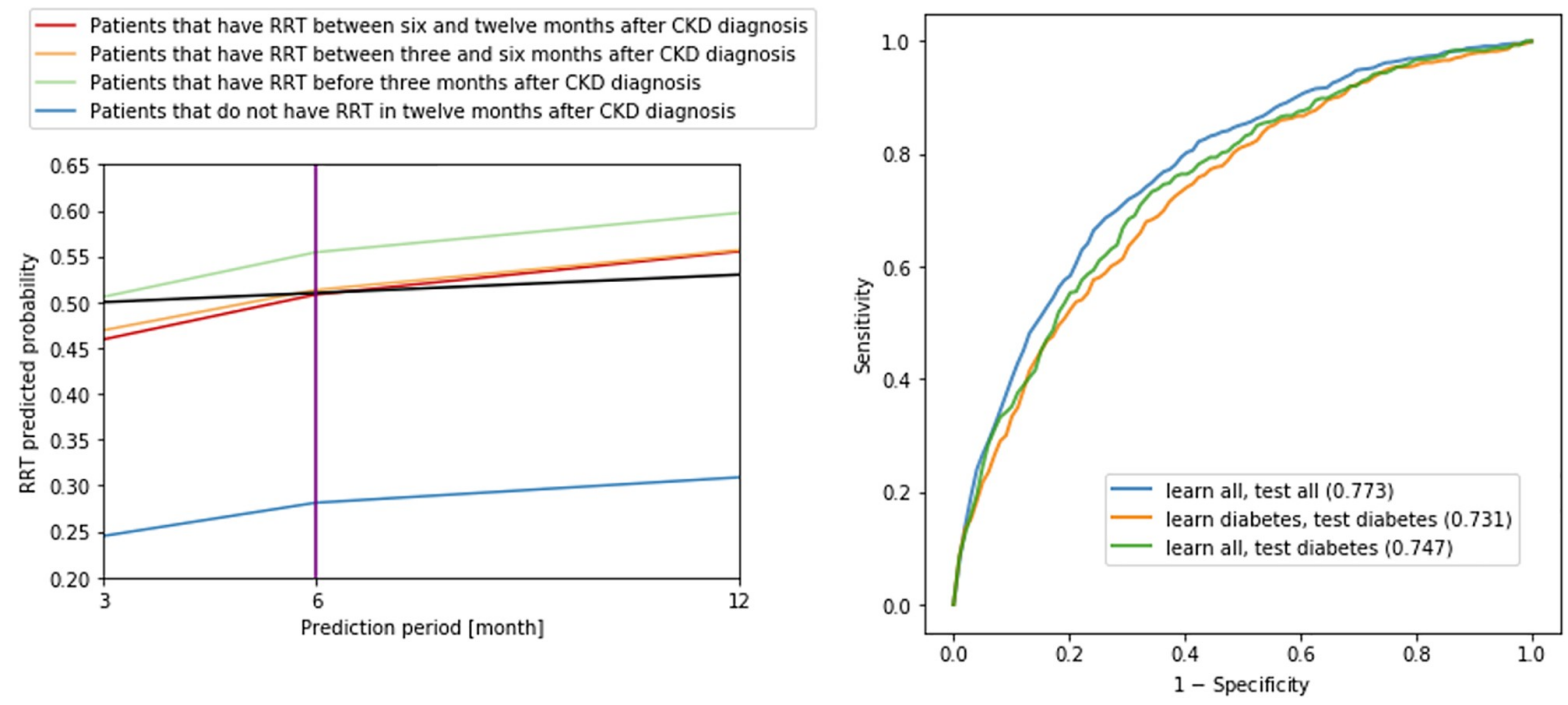
Left: Predicted probabilities of needing RRT, averaged at each prediction period. Blue, red, orange and green lines show the average predicted probabilities of needing RRT for 4 distinct sets of patients as described in the legend. Three prediction periods are shown (12, 6 and 3 months), where, for each period, one Logistic Regression model is used. The black line shows the ideal threshold for all three models. More precisely, using this threshold, all the models (on average) correctly predict RRT for all subsets of patients. Right: AUCs obtained with Logistic Regression for various tests. The AUC values are shown in brackets.

The previous analysis focused on classification of patients into those that will need RRT and those that will not need it. However, ML models, including Logistic Regression are also able to return the probabilities of needing RRT. We analyzed the probability of needing RRT of the same patient at various prediction intervals, where, at each interval, a different Logistic Regression model was applied. The predicted probabilities were further averaged for each prediction interval and the obtained results are shown in Fig 6. This figure shows that the ideal thresholds for prediction periods can be selected at (average) probabilities:

- between 0.470 and 0.505 for prediction period of three months,
- between 0.509 and 0.513 for prediction period of six months, and
- between 0.310 and 0.555 for prediction period of twelve months.

The black line in this figure shows the following thresholds: 0.50 for three months, 0.51 for six months and 0.53 for twelve months. At these average probabilities, all models produce the best results, since all patients are on average classified correctly by all three models. More precisely, the line of patients with RRT before three months is always above the threshold, which is correct; the line of patients with RRT between three and six months is above the threshold at six and twelve months, but below threshold at three months, which is correct; the line of patients with RRT between six and twelve months is above threshold at twelve months, but below threshold at three and six months, which is correct; and the line of patients without RRT is always below the threshold, which is also correct.

### Comparing models on all the patients with models on diabetes patients

Diabetic nephropathy is a common complication of diabetes. Since diabetes patients are a more homogeneous group than all CKD patients, it might be easier to predict RRT on CKD patients with diabetes only. To test this hypothesis, we preprocessed the data with filtering in order to select only patients with diabetes type 2 or type undefined. Afterward, we performed three tests:

- We built and tested the models on all the patients.
- We built and tested the models on only patients who had diabetes prior to the CKD diagnosis.
- We built the models on all the patients, but tested them only on the patients who had diabetes prior to the CKD diagnosis.

Note that in all three tests, stratified 10-fold cross-validation was applied. In addition, the selected model was Logistic Regression, the prediction interval was twelve months, data balancing was used, but there was no feature selection or dimensionality reduction. In the third test, data of all the patients was used, but during model evaluation only patients with diabetes were selected from the test set. The results are shown in Table 5 and Fig 6. In addition, the confusion matrices for all three tests are shown in S10 Table–S12 Table in S1 File.

**Table 5:** Performance metrics for various tests.

Test	No patients	AUC	Sensitivity	Specificity
Learn all Test all	8492	0.773	0.623	0.781
Learn diabetes Test diabetes	3104	0.731	0.555	0.780
Learn all Test diabetes	7952	0.747	0.696	0.689

The results show that the highest AUC is obtained when no filtering is applied, i.e., when we learn and test on all the patients. The second highest AUC is obtained when learning on all the patients, but testing on only patients with diabetes. The lowest AUC is obtained when learning and testing on patients with diabetes. This suggests that prediction for patients with diabetes is a bit harder than average, and that having more training data (8,492 in total vs. 3,104 with diabetes) is more important than having more homogeneous data.

The confusion matrices show that when we learn on all the patients, better classification is obtained for those patients that have RRT. On the other hand, when we used diabetes patients for learning, better classification is obtained for those patients that do not have RRT.

## Discussion

In this retrospective study, we provided a novel approach of screening CKD patients to predict the chances of future RRT based on the clinical data using ML algorithms. A disease-specific subset, i.e., data based on the patients’ diagnosis and RRT occurrences between 1998 and 2011, was analyzed from the NHIRD dataset [25]. Although the results are not yet as such suitable for adoption into clinical practice for individual patients, the study provides a strong basis and a variety of approaches for future studies of forecasting models in healthcare.

There exists other work aimed at predicting RRT, but it used laboratory data, which is not as readily available as the information on comorbidities used in this paper. For example, Lee et al. analyzed electronic medical records of 4,500 patients from South Korea from 1997 to 2012 in order to predict the time of RRT [16]. For this purpose, they used six months of 12 clinical data variables such as serum albumin, serum hemoglobin, serum phosphorus, serum potassium, and eGFR, in addition to demographic variables, and five comorbidities. The applied ML model was Multivariate Cox proportional hazards regression. The AUC on the test set was between 0.80 and 0.86, depending on the CKD stage. Evans et al. analysed the relationship between death and RRT on one hand, and age, sex, primary renal disease, body mass index, and glomerular filtration rate (GFR) on the other hand [17]. The results showed that GFR at entry was clearly linked to the incidence of RRT, and age was related inversely to incidence of RRT. Lea et al. analyzed the data of 1,094 patients and the results showed that the change in urinary protein level represents a predictor for progression to RRT [18]. More precisely, the early change in urinary protein level from baseline to the 6-month point resulted in the subsequent rate of decline in GFR and the incidence of end-stage CKD. Iseki et al. applied logistic regression on data from 107,192 patients from Okinawa to predict end-stage CKD [19]. The analysed variables included sex, age at screening, proteinuria, hematuria, systolic and diastolic blood pressure. The results showed that proteinuria was the most potent predictor, and the next most potent predictor was hematuria. Norouzi et al. used clinical and laboratory data on 465 CKD patients for predicting renal failure progression in CKD was conducted by covering the clinical data from 2002 to 2011 [20]. The model was built using Adaptive Neuro-Fuzzy Inference System (ANFIS) and its results showed reliable accuracy for Glomerular Filtration Rate (GFR) prediction in the time periods of 6, 12 and 18 months.

Similarly, there are various studies in the literature that have harnessed the potentials of ML algorithms for the development of Acute Kidney Injury prediction models using EMRs. For example, Tomašev et al. analyzed data of 703,782 patients and built a neural network model for predicting acute kidney injuries that required subsequent administration of dialysis within 48 hours [21]. The input data consisted of procedures, diagnoses, prescriptions, laboratory tests, vital signs, and admissions. Park et al. predicted the occurrence of acute kidney injury (AKI) in cancer patients using the laboratory measurements [22]. A similar experiment with similar (laboratory) input data was also conducted by Mohamadlou et al., which aimed at predicting AKI within 12, 24, 48 and 72 hours [23], and Zhang et al., which applied ML for differentiating between patients who would and would not respond to fluid intake in urine output in AKI [24].

Looking at the most common comorbidities in the dataset, those found in more than 10% of the patients were Diabetes Mellitus Type II (in 32% of the patients), Essential Hypertension (45%), and Hypertensive Heart Disease (20%). A more detailed statistics on the comorbidities is available in S13 Table. We next analysed the comorbidities found to be most relevant in our experiment (shown in Fig 5) and found them to be mostly in line with the knowledge about CKD. The presence of pleural effusion and lung edema could be due to CKD or an interplay of CKD and coronary artery disease (CAD). Most of these patients have hypoproteinemia, a strong predictor of progressive kidney disease. Hypoproteinemia is especially common in patients with chronic glomerulonephritis and diabetic nephropathy. Most patients with CKD resulting from progression of chronic glomerulonephritis or diabetes mellitus have third space collections as a consequence of hypoproteinemia and low GFR, one such collection being pleural effusion. Normocytic normochromic anemia is seen in CKD patients with eGFR less than 60 mL/min/1.73 m2 [43]. Therefore, it is reasonable that these features play a prominent role in the model. However, it is surprising to find chronic liver disease and cirrhosis as predicting coefficients. The only way this can be justified could be due to the presence of an unusually high prevalence of these diseases in our study group.

There were several limitations and barriers associated with our current study. Since our dataset was extracted from NHIRD (and is thus representative of the data that would be available to an insurance company or a hospital manager), the source does not contain patients’ personal characteristic, such as height, weight, and lifestyle. The dataset also lacks crucial laboratory data like eGFR and albuminuria needed to categorize the stage of CKD [44], as well as it is missing detailed laboratory test results and medical notes. Such data would be beneficial for a model that can be used in clinical decision-making, and could also allow the patients to prepare for the treatment in advance. However, if we used such additional information, the model would no longer be so suitable for low- and medium-income countries where this information may not be available. Due to the retrospective nature of our study, there could be chances of selection and misclassification bias. In addition, the scope of extracted dataset from NHIRD was limited, as the majority of the population was Taiwanese. In order to apply our proposed approach in generalized context, a large scale validation with heterogeneous population is recommended. We suggest that this kind of broader level investigations should be performed first for re-evaluating the performance and consistency of our proposed approach before inclusion in clinical practices. If adopted in practice, our approach can support clinicians in treatment plan development and decision making process for initiating the hemodialysis at the right time for CKD patients. In addition, it can also allow the patients to pool up the financial resources if they need to fund the treatment themselves.

## Conclusion

The main advantage of the existing work is in the use of comorbidities for prediction of RRT. A large heterogeneous population should be used to create and evaluate the model’s performance before it can be applied to clinical practice. It must be understood that by using ML algorithms, our study provides a screening approach for predicting the chances of upcoming RRT based on the clinical data, therefore this should neither be considered as clinical guideline nor a diagnostic / therapeutic tool for CKD patients. On the other hand, the results at this point are more interesting from the point of view of policy-makers, such as hospital managers or health officials, or insurance companies. Using predictive models on a general population with the data available can allow for better planning and allocation of resources. Future scope lies in coming up with a prediction model that would factor in the more clinical data (use of specific drugs / associated comorbidities / dietary interventions / degree of blood pressure control / degree of blood sugar control) in predicting the outcomes and providing a possible chance for us to tailor the therapeutic interventions accordingly.

We need to realise that the ML algorithms our study provides need to be considered as a possible screening tool to predict the time frame of progression of CKD patient before he/she would need RRT.

## Supporting information

S1 File (PDF)

S2 File (TEX)
