# Supplementary material for: Using machine learning models to predict the initiation of renal replacement therapy among chronic kidney disease patients
Source: PLoS One. 2020 Jun 5;15(6):e0233976. doi: 10.1371/journal.pone.0233976 (PMC7274378; doi:10.1371/journal.pone.0233976)
Supplement: S1 File — (PDF) [file pone.0233976.s001.pdf]

Using machine learning models to predict the initiation of renal replacement therapy among chronic kidney disease patients

Erik Dovgan[1*], Anton Gradišek[1], Mitja Luštrek[1], Mohy Uddin[2], Aldilas Achmad Nursetyo[3], Sashi Kiran Annavarajula[4], Yu-Chuan Li[3], Shabbir Syed-Abdul[3*]

1 Jožef Stefan Institute, Department of Intelligent Systems, Jamova cesta 39, SI-1000 Ljubljana, Slovenia
2 Executive Office, King Abdullah International Medical Research Center, King Saud bin Abdulaziz University for Health Sciences, Ministry of National Guard – Health Affairs, Riyadh, Kingdom of Saudi Arabia
3 Taipei Medical University, Graduate Institute of Biomedical Informatics, College of Medical Science and Technology, Taipei, Taiwan
4 Yashoda Hospitals, Department of Nephrology, Malakpet, Hyderabad, India

* erik.dovgan@ijs.si, drshabbir@tmu.edu.tw

Supporting information

The tables in this section show:

- Table S1-Table S5: Statistics of AUC for each feature selection approach, data preprocessing and ML algorithm independently when predicting twelve months ahead.
- Table S6-Table S7: Top 10 algorithms in combination with data processing, when predicting six- and three- months ahead respectively.
- Table S8-Table S9: Confusion matrices for six- and three- months-ahead prediction using Logistic Regression.
- Table S10-Table S12: Confusion matrices for models that are built and tested on data of all patients or of patients with diabetes only.
- Table S13: Statistics on the comorbidities in the dataset, including RRT and hazard ratio (HR) for each prediction period.

Table S1. AUCs of approaches for obtaining features.

	Mean	Std	Min	25 %	50 %	75 %	Max
Raw	0.676	0.066	0.561	0.625	0.694	0.705	0.765
Percentage	0.642	0.137	0.321	0.575	0.686	0.746	0.769
Boolean	0.685	0.074	0.561	0.631	0.702	0.746	0.767
Time	0.665	0.097	0.497	0.595	0.693	0.752	**0.773**

Table S2. AUCs of feature selection approaches.

	Mean	Std	Min	25 %	50 %	75 %	Max
None	0.676	0.066	0.561	0.625	0.694	0.705	**0.765**
Correlations	0.573	0.014	0.549	0.560	0.576	0.586	0.587
Comorbidities	0.568	0.056	0.498	0.527	0.557	0.619	0.657
SHL	0.552	0.023	0.509	0.535	0.563	0.568	0.569

Table S3. AUCs of filtering approaches.

	Mean	Std	Min	25 %	50 %	75 %	Max
None	0.676	0.066	0.561	0.625	0.694	0.705	**0.765**
Diabetes	0.650	0.059	0.544	0.616	0.656	0.689	0.732

Table S4. AUCs of dimensionality reduction approaches.

	Mean	Std	Min	25 %	50 %	75 %	Max
None	0.676	0.066	0.561	0.625	0.694	0.705	**0.765**
PCA	0.649	0.057	0.548	0.626	0.644	0.681	0.729

Table S5. AUCs of ML algorithms.

	Mean	Std	Min	25 %	50 %	75 %	Max
Decision tree	0.577	0.037	0.512	0.549	0.580	0.602	0.660
Bagging Decision Tree	0.629	0.065	0.526	0.560	0.641	0.690	0.707
Random Forest	0.620	0.056	0.527	0.561	0.627	0.670	0.697
XGBoost	0.691	0.060	0.568	0.654	0.698	0.736	0.767
SVM	0.613	0.135	0.269	0.594	0.635	0.691	0.762
SGD	0.673	0.064	0.527	0.643	0.671	0.731	0.768
Nearest Neighbors	0.582	0.029	0.512	0.566	0.581	0.602	0.632
Naive Bayes	0.574	0.070	0.498	0.511	0.554	0.651	0.696
Logistic Regression	0.687	0.059	0.569	0.649	0.675	0.739	**0.773**
Neural Network	0.639	0.053	0.538	0.596	0.662	0.672	0.706

Table S6. Top 10 algorithms in combination with data processing, when predicting six months ahead. Results are sorted with respect to AUC.

Model	Features	Balance	AUC	Sensitivity	Specificity
Logistic Regression	time	no	0.791	0.059	0.995
SGD Classifier	time	no	0.790	0.055	0.995
XGBoost	raw	no	0.785	0.021	0.999
XGBoost	boolean	no	0.782	0.018	0.999
XGBoost	percentage	no	0.781	0.016	0.999
SGD Classifier	time	yes	0.780	0.630	0.794
XGBoost	time	no	0.778	0.017	0.999
Logistic Regression	time	yes	0.778	0.568	0.832
XGBoost	raw	yes	0.778	0.562	0.834
Logistic Regression	percentage	no	0.777	0.020	0.998

Table S7. Top 10 algorithms in combination with data processing, when predicting three months ahead. Results are sorted with respect to AUC.

Model	Features	Balance	AUC	Sensitivity	Specificity
SGD	time	no	0.801	0.017	0.999
Logistic Regression	time	no	0.798	0.031	0.998
XGBoost	time	no	0.793	0.008	1.000
XGBoost	raw	no	0.792	0.008	0.999
XGBoost	boolean	no	0.789	0.012	1.000
XGBoost	percentage	no	0.788	0.004	1.000
Logistic Regression	percentage	no	0.784	0.002	0.999
SGD	percentage	no	0.784	0.002	0.999
XGBoost	boolean	yes	0.782	0.548	0.847
SGD	percentage	yes	0.781	0.514	0.838

Table S8. Confusion matrix for six-months-ahead prediction.

		Predicted	
		No	Yes
True	No	10351	2087
	Yes	328	432

Table S9. Confusion matrix for three-months-ahead prediction.

		Predicted	
		No	Yes
True	No	13639	2170
	Yes	256	260

Table S10. Confusion matrix for the model that was built on data of all patients and tested on data of all patients.

		Predicted	
		No	Yes
True	No	5819	1628
	Yes	394	651

Table S11. Confusion matrix for the model that was built on data of patients with diabetes and tested on data of patients with diabetes.

		Predicted	
		No	Yes
True	No	2028	570
	Yes	225	281

Table S12. Confusion matrix for the model that was built on data of all patients and tested on data of patients with diabetes.

		Predicted	
		No	Yes
True	No	1789	809
	Yes	154	352

Table S13. Statistics on the comorbidities in the dataset, including RRT and hazard ratio (HR) for each prediction period.

Comorbidity	No. cases	One year				Six months				Three months			
		No. cases	With RRT	Without RRT	HR	No. cases	With RRT	Without RRT	HR	No. cases	With RRT	Without RRT	HR
All patients	19954	8492	1045	7447		13198	760	12438		16325	516	15809	
Diabetes	6506 (33%)	3123 (37%)	508 (49%)	2615 (35%)	1.4	4545 (34%)	357 (47%)	4188 (34%)	1.4	5479 (34%)	231 (45%)	5248 (33%)	1.3
Diabetic Type I	198 (1%)	108 (1%)	27 (3%)	81 (1%)	2.4	149 (1%)	22 (3%)	127 (1%)	2.8	173 (1%)	12 (2%)	161 (1%)	2.3
Diabetic Type II	5928 (30%)	2863 (34%)	480 (46%)	2383 (32%)	1.4	4132 (31%)	338 (44%)	3794 (31%)	1.5	5001 (31%)	222 (43%)	4779 (30%)	1.4
Diabetic Type II unspecified	6468 (32%)	3104 (37%)	506 (48%)	2598 (35%)	1.4	4515 (34%)	356 (47%)	4159 (33%)	1.4	5446 (33%)	230 (45%)	5216 (33%)	1.4
Essential hypertension	9052 (45%)	4622 (54%)	633 (61%)	3989 (54%)	1.1	6370 (48%)	452 (59%)	5918 (48%)	1.2	7640 (47%)	300 (58%)	7340 (46%)	1.3
Hypertensive heart disease	3938 (20%)	2124 (25%)	271 (26%)	1853 (25%)	1.0	2844 (22%)	195 (26%)	2649 (21%)	1.2	3350 (21%)	128 (25%)	3222 (20%)	1.2
Hypertensive chronic kidney disease	250 (1%)	139 (2%)	27 (3%)	112 (2%)	1.7	181 (1%)	18 (2%)	163 (1%)	1.8	211 (1%)	10 (2%)	201 (1%)	1.5
Hypertensive heart and chronic kidney disease	175 (1%)	112 (1%)	17 (2%)	95 (1%)	1.3	136 (1%)	13 (2%)	123 (1%)	1.7	155 (1%)	12 (2%)	143 (1%)	2.6
Secondary hypertension	159 (1%)	101 (1%)	23 (2%)	78 (1%)	2.1	120 (1%)	16 (2%)	104 (1%)	2.5	138 (1%)	12 (2%)	126 (1%)	2.9
Acute glomerulonephritis	110 (1%)	61 (1%)	16 (2%)	45 (1%)	2.5	81 (1%)	10 (1%)	71 (1%)	2.3	95 (1%)	9 (2%)	86 (1%)	3.2
Chronic glomerulonephritis	1727 (9%)	1120 (13%)	232 (22%)	888 (12%)	1.9	1351 (10%)	177 (23%)	1174 (9%)	2.5	1522 (9%)	118 (23%)	1404 (9%)	2.6
Acute and chronic glomerulonephritis	1811 (9%)	1159 (14%)	241 (23%)	918 (12%)	1.9	1408 (11%)	182 (24%)	1226 (10%)	2.4	1591 (10%)	123 (24%)	1468 (9%)	2.6
Polycystic kidney	65 (0%)	43 (1%)	12 (1%)	31 (0%)	2.8	51 (0%)	7 (1%)	44 (0%)	2.6	59 (0%)	3 (1%)	56 (0%)	1.6
Nephritis NEC	16 (0%)	11 (0%)	4 (0%)	7 (0%)	4.1	15 (0%)	2 (0%)	13 (0%)	2.5	16 (0%)	2 (0%)	14 (0%)	4.4
Calculus of kidney and ureter	1228 (6%)	486 (6%)	32 (3%)	454 (6%)	0.5	726 (6%)	26 (3%)	700 (6%)	0.6	951 (6%)	12 (2%)	939 (6%)	0.4
Calculus of lower urinary tract	70 (0%)	23 (0%)	0 (0%)	23 (0%)	0.0	34 (0%)	0 (0%)	34 (0%)	0.0	51 (0%)	0 (0%)	51 (0%)	0.0
Urinary obstruction	19 (0%)	8 (0%)	1 (0%)	7 (0%)	1.0	13 (0%)	1 (0%)	12 (0%)	1.4	14 (0%)	0 (0%)	14 (0%)	0.0
Vesicoureteral reflux	5 (0%)	1 (0%)	0 (0%)	1 (0%)	0.0	2 (0%)	0 (0%)	2 (0%)	0.0	2 (0%)	0 (0%)	2 (0%)	0.0
Infections of kidney	455 (2%)	209 (2%)	27 (3%)	182 (2%)	1.1	284 (2%)	23 (3%)	261 (2%)	1.4	362 (2%)	16 (3%)	346 (2%)	1.4
